# Experimental Method for Identifying Regions of Use of a Progressive Power Lens Using an Eye-Tracker: Validation Study

**DOI:** 10.3390/life14091178

**Published:** 2024-09-19

**Authors:** Clara Benedi-Garcia, Pablo Concepcion-Grande, Eva Chamorro, Jose Miguel Cleva, José Alonso

**Affiliations:** Clinical Research Department, Indizen Optical Technologies, 28002 Madrid, Spain; evachamorro@iot.es (E.C.); jmcleva@iot.es (J.M.C.); jalonso@iot.es (J.A.)

**Keywords:** regions of use, pupil position, eye-tracking, progressive power lenses

## Abstract

Power distribution of progressive power lenses provides usable regions based on power distribution analysis. However, recent studies demonstrated that these regions are not always used for certain tasks as predicted. This work determines the concordance between the actual region of lens use and compares it with the theoretically located regions. The pupil position of 26 subjects was recorded using an eye-tracking system (Tobii-Pro-Glasses 3) at distance and near-reading tasks while wearing a general use progressive power lens. Subjects were asked to read aloud a text showed on a screen placed at 5.25 m and 37 cm while looking though the central and lateral regions of the lens. The pupil position was projected onto the back surface of the lens to obtain the actual region of use for each fixation. Results showed that the actual region of use matched with the theoretically located. On average, the concordance between the actual and theoretical regions of use was 85% for a distance-reading task and 73% for a near-reading task. In conclusion, the proposed method effectively located the actual regions of the lens used, revealing how users’ posture affects lens usage. This insight enables the design of more customized progressive lenses based on the areas used during vision-based tasks.

## 1. Introduction

Presbyopia is an age-associated condition that reduces the ability to focus on near objects, causing blurred vision at near distances. It is a natural aspect of aging that typically manifests around the age of 40 [1]. Progressive power lenses (PPLs) have gained popularity among presbyopes as an effective solution, since they provide a smooth transition of spherical power from distance to near vision, enabling wearers to achieve clear vision at all distances by adjusting their gaze direction [2]. However, this power variation along the vertical axis results in unwanted astigmatic and spherical power fluctuations in the lateral regions of the lens, which produces distortion, blurring, and the swim effect [3] and limits the useful visual areas impacting the quality of vision [4,5]. The relationship between the useful visual areas and the unwanted astigmatism is determined by the characteristics of the PPL design, which varies depending on the manufacturer and model.

The analysis of power distribution maps of a PPL design provides useful metrics to characterize this type of lens. However, they do not represent the visual perception of users, which varies depending on each subject [5,6,7]. Some of the methods proposed to assess the visual perception with PPLs involve the analysis and representation of power distribution maps generated using lens mappers [8] or calculated through raytracing to obtain user-perceived power distribution maps [9]. These approaches are based on geometric calculations to estimate fields of view [6,7,8,10]. For this reason, we can also find in the bibliography numerous studies that have evaluated visual performance with PPLs by means of satisfaction questionnaires [11] or optometric tests such as visual acuity (VA) or contrast sensitivity [12,13].

On the other hand, several studies have employed eye-tracker techniques for the assessment of visual performance with PPLs. Video-based eye-tracker systems use infrared light reflection images from the cornea and pupil to calculate gaze position [14,15]. An infrared light source projects light onto the eye, and a camera captures the reflections. The analysis focuses on the first Purkinje image, originating from the cornea’s anterior surface, and the pupillary center to determine gaze position. As the eye moves, the pupillary center shifts spatially, while the first Purkinje image remains stable. Analyzing position variations between the pupillary center and the first Purkinje image allows for precise determination of gaze position and direction [16]. Eye-tracker systems provide useful information about the eye movement patterns while wearing PPLs during different tasks. Concepcion-Grande et al. [17] analyzed the reading performance using PPLs, and they found that visual performance in terms of reading time, total duration of fixations, and fixation count correlated with the working distance and the evaluated PPL. As another example, Rifai et al. [18] studied eye movement coordination during urban driving with and without PPLs, obtaining that the use of PPLs requires a coordination between gaze position and head position facing driving stimuli.

While these studies furnish valuable insights into how the power distribution of PPLs impacts user satisfaction and eye movements during specific tasks, they fall short of detailing the specific regions of the lens employed in these activities. Sheedy et al. [19] identified important distinguishing characteristics of PPLs based on power distribution, which have traditionally been used to compare different designs. Recent studies have shown that the Sheedy regions are not always used at certain working distances [20]. Recognizing how individuals make use of different regions of these lenses becomes essential to maximizing the potential of manufacturing technologies and refining lens designs. In this sense, the gaze data can be processed by advanced algorithms to calculate the point of intersection of the gaze direction vector with any plane, such as the plane of a lens positioned in front of the user’s eye [21]. The points on the lens through which visual axes pass when focusing on an object during a specific visual task are called viewing-through points. The locus of viewing-through points occurring during the time span of fixations while performing a visual task is defined as the region of use for that visual task. The regions of use of the lens can be used to understand the postural habits of PPL users during the execution of specific tasks, and it would help to understand how wearers use this type of lens.

For this reason, in this study we tested a method involving an eye-tracker device that identifies the actual lens regions being used while performing different visual tasks from the records of pupil position and gaze direction obtained by an eye-tracker. The goal of this study is to determine the concordance between the region of use of the lens identified by the proposed experimental method and the theoretically calculated regions of use of a PPL. It is expected to find a match between the theoretical regions of use of progressive power lenses and the actual regions of use during distance and near reading tasks, with a higher concordance for the on-axis conditions and the vertical components.

## 2. Materials and Methods

### 2.1. Study Design

A prospective observational longitudinal study was conducted to assess the vertical and horizontal region of use on the back surface of the lens during a reading test, for both distance and near vision. The study adhered to the principles outlined in the Declaration of Helsinki. Complete approval for the study was granted by the Complutense University of Madrid Ethics Committee’s Review Board (CE_20210715-3_SAL). Prior to the commencement of the study, all participants provided written informed consent. At the conclusion of the study, participants were rewarded with a pair of glasses with PPLs.

### 2.2. Participants

The sample consisted of presbyopic participants of both genders over the age of 45, who had at least 6 months prior experienced wearing PPLs. The inclusion criteria were: (1) refractive error of −6 D to +4 D with astigmatism lower than or equal to 2.5 D; (2) a near addition range of +1 D to +3 D; (3) monocular visual acuity better than 0.10 logMAR and binocular visual acuity better than 0.0 logMAR; and (4) anisometropia lower than 1.5 D. Subjects were excluded if they had any medical diseases that could affect vision, binocular vision anomalies, ocular pathologies, or if they were undergoing any pharmacological treatment that could affect the visual function. The sample size was determined using data from a pilot study carried out with five participants who met the same inclusion criteria as described above. The calculation was performed using a GRANMO version 7.12 sample size calculator (Institut Municipal d’Investigació Mèdica, Barcelona, Spain). The predicted sample size was 27 subjects, with an assumed alpha risk of 0.05 and a beta risk of 0.1 in two-tailed testing. Also, it was considered to have a 15% dropout rate.

### 2.3. Procedure

All subjects were subjected to an optometric examination to determine whether they meet the inclusion criteria. The optometric examination consisted of VA testing with the PVVAT test (Precision Vision, La Salle, III), subjective refraction at distance and near vision, stereoacuity evaluation with the Titmus test, cover test, Worth test, and ocular motion analysis. After optometrists determined that the participant met the inclusion criteria, fitting parameters and position of wear (pupillary distance, pupil height, pantoscopic tilt, back vertex distance, and frame wrap angle) for the wearable eye-tracker were measured, and PPL lenses were ordered. The eye-tracker system used allows the positioning of ophthalmic lenses in a manner very similar to their usual use and allowing the cameras to directly record the pupils. Recordings of pupil position and size at the different working distances were acquired during a one-hour independent visit with two-minute breaks between each measurement to minimize visual fatigue.

### 2.4. Progressive Power Lenses

**Figure 1:**
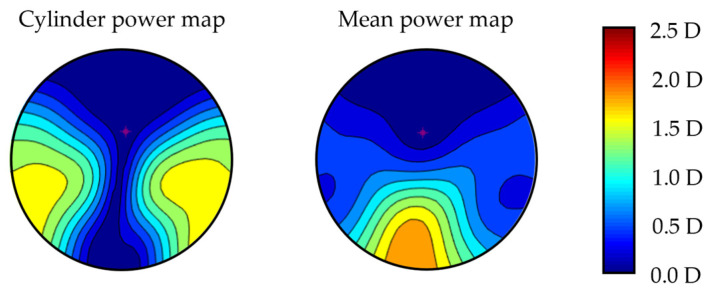
Cylinder and mean power map distribution for plano addition 2 D prescription using standard parameters of the PPL used in this research.

**Figure 2:**
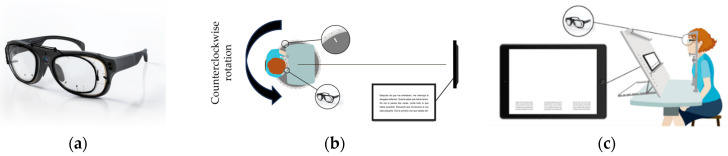
Image of the clip-on with the prescription lenses attached to the front part of the eye-tracker Tobii Glasses PRO 3 (**a**). Scheme of the distance-reading task using eye-tracker (**b**). Scheme of the near reading task using eye-tracker (**c**).

A balanced personalized free-form PPL design (Endless Steady Balance, IOT, Madrid, Spain) was used considering distance prescription, near prescription, fitting parameters, and position of wear of the eye-tracker glasses for each participant, and all of them had a length corridor of 16 mm. Cylinder power and mean power map distribution for a plano addition 2 D prescription considering standard position of wear parameters are shown in Figure 1. The lenses were mounted in a specially designed clip-on, which was attached to the front part of the eye-tracker system (Figure 2a).

### 2.5. Experimental Evaluation of Regions of Use by Eye-Tracker

A wearable eye-tracker device (Tobii-Pro Glasses 3, Tobii AB, Danderyd, Sweden) with a sample rate of 50 Hz was employed to record binocular pupil position. Recordings were conducted during both distance and near reading tasks including only subjects whose recordings had a data loss lower than 10% [15,22].

For distance-reading task, subjects read aloud a text displayed on a screen monitor (Asus LCD Monitor VP228HE 21.5″, Asus, Taiwan) positioned 5.25 m away and 31.7 cm above their primary gaze position. The used texts were part of a set of nine paragraphs, which were randomized for each participant. The text had an angular dimension of 4.2° horizontally and 2.3° vertically, and it was composed of five text lines with a font size of 0.4 logMAR. To evaluate different viewing positions, subjects were rotated counterclockwise into three different orientations: one on-axis and two off-axis viewing positions (10° and 15°). Off-axis viewing positions were achieved using a rotation platform with a chinrest to restrict head movement (Figure 2b).

For the near-reading task, subjects read aloud a text located 0.37 m away using a set of three different texts, which were randomized for each participant. These texts were displayed on a screen (Microsoft Surface PRO 4, 12.3″, Microsoft, Redmond, WA, USA) and each one was composed of three columns of five text lines with a font size of 0.4logMAR. The text had an angular dimension of 28° horizontally and 3.6° vertically. The central column was centered with the participant’s midline, and each of the lateral columns was 12.5° to the left side and 12.5° to the right side, measured from the center of the columns. The horizontal extension of each column was 3°. To assess different viewing positions, a tablet with a chinrest was used to restrict head movement (Figure 2c).

The texts used for each reading condition were linguistically analyzed to ensure they were similar in terms of word count, syllables, single-syllable words, mean characters per word, and ISFZ index [23,24] (Table 1). Additionally, a pilot study with 10 emmetropic subjects with normal vision and without wearing lenses was carried out to ensure there were no differences between each text for each distance in terms of reading time, total duration of fixations, and fixation count.

**Figure 3:**
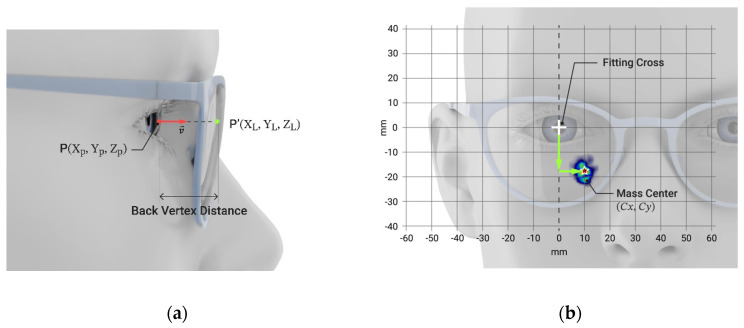
Schema of the calculation process for the projection of the gaze direction on the back surface of the lens (P’(X_L_,Y_L_,Z_L_)) considering the pupil position (P’(X_P_,Y_P_,Z_P_)) (**a**). Diagram of the vertical and horizontal region of use for right eye looking at near-reading right side, considering the lens viewed from the front, the center of mass ((C_x_,C_y_), depicted by the star), and the center of reference of the eye tracker system (**b**).

For each reading condition, the region of use of the PPL during the tasks was obtained through the calculation of the intersection points of the direction of sight with the back surface of the lens, considering all points where the sight direction intersects the lens surface for each recording. An algorithm was developed to project the pupil position onto the back surface of the lens for each fixation [21]. From the eye-tracker device, the gaze direction coordinates ($\vec{v}$) and the pupil position P (X_p_, Y_p_, Z_p_) were obtained. Knowing a point and a direction, it is possible to calculate the intersection point P’ (X_L_, Y_L_, Z_L_) of the direction of sight with the back surface of the lens (Figure 3a). In addition, some frame parameters were needed to establish the location of the fixations with respect to the frame, and thus, with respect to the lens. In particular, pupil height, back vertex distance, and interpupillary distance were considered for the calculation.

The metrics of the study were defined from the fixations on the plane of the back surface of the lens. The vertical and horizontal regions of use (Cx, Cy) were defined as the vertical and horizontal components of the mass center of the fixations with respect to the fitting cross of the lens (Figure 3b), measured in millimeters, and considering the lens viewed from behind.

### 2.6. Theoretical Estimation of Zones of Use

**Figure 4:**
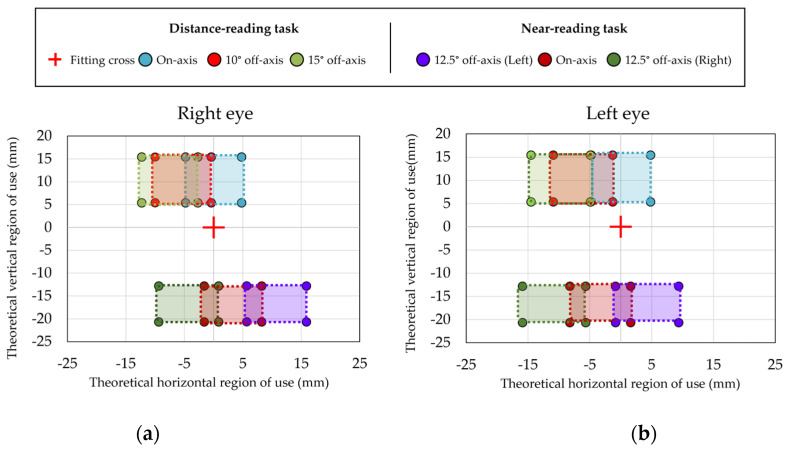
Theoretical regions of use of the lens that should be used according to the condition at far (distance-reading on-axis, 10° off-axis, and 15° off-axis) or near (12.5° off-axis (Left), on-axis, and 12.5° off-axis (Right)) for right eye (**a**) and left eye (**b**). The red cross represents the fitting cross (FC).

The theoretical regions of use were calculated using a geometrical method considering the distances between center of rotation of the eye with the head fixed and the target test (5.25 m and 0.37 m for distance and near, respectively) as well as the different viewing positions at distance-reading (on-axis, 10° off-axis and 15° off-axis) and near-reading (12.5° off-axis (Left), on-axis and 12.5° off-axis (Right)). The regions of use on the lens were calculated based on the real size of the texts on the back surface of the lens and considering the average pupil diameter tolerance. Averaged pupil diameter was 3.73 mm at distance-reading tasks and 2.88 at near-reading tasks. In Figure 4, the theoretical locations of regions of use on the back surface of the lens are illustrated.

### 2.7. Statistical Analysis

Mean and standard deviation of the region of use, determined by the experimental method, was calculated for all conditions, including on and off-axis viewing position as well as distance and near reading task. Analysis of concordance between the regions of use was determined experimentally and theoretically, considering the percentage of fixations for all subjects identified by the experimental method during the reading tasks were located within the theoretical regions of use.

Additionally, a descriptive analysis of means and standard deviation was conducted to analyze expected differences in horizontal and vertical regions of use between different viewing positions for distance and near reading tasks.

## 3. Results

### 3.1. Sample Characteristics

The sample was composed of 26 participants (17 men and 9 women) with a mean age of 55 ± 7 years (ranging from 46 to 64 years). According to the optometric examination, the average refractive error was −0.83 ± 2.26 D (ranging from −5.34 to 2.25 D). There were 14 myopic participants, 8 hyperopic participants, and 4 emmetropic participants. The participants’ addition power ranged from 1 D to 2.5 D, with an average value of 2.00 ± 0.44 D. Of the 26 participants included in the study, 25 of them fulfilled the data quality criteria for the DR task and 16 of them met the criteria for the near-reading task.

### 3.2. Distance-Reading Task

**Figure 5:**
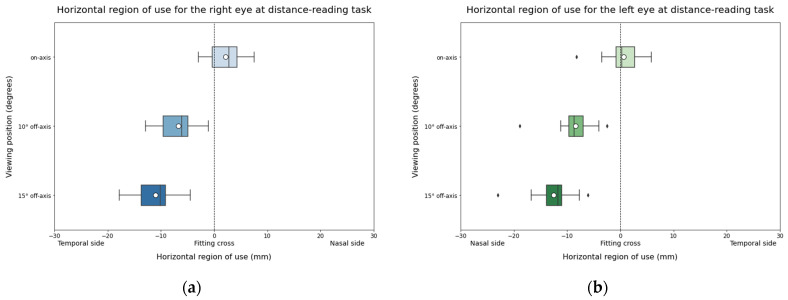
Horizontal region of use for the left eye (**a**) and the right eye (**b**) during distance-reading task. The solid black line represents the median and the circle represents the mean for each box.

Regarding the analysis of the horizontal region of use, horizonal variations towards the nasal side for the left eye and towards the temporal side for the right eye were expected when participants looked through off-axis viewing positions compared to the centered viewing position due to the participant’s counterclockwise rotation and fixed head and screen. Specifically, at 10° and 15° off-axis positions, the left eye shifted towards nasal values (Figure 5a), while the right eye shifted towards temporal values compared to the on-axis position, with a more pronounced effect at 15° (Figure 5b).

**Figure 6:**
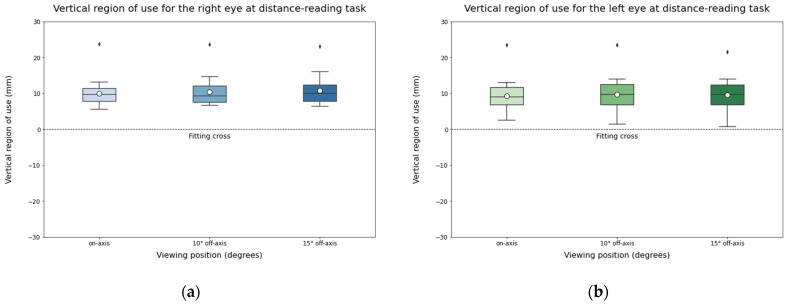
Vertical region of use for the left eye (**a**) and the right eye (**b**) during distance-reading task. The solid black line represents the median and the circle represents the mean for each box.

For the vertical region of use, since the test and the participants’ head maintained in a stable vertical region of use across different viewing positions, it was not expected to find differences between them (Figure 6).

**Figure 7:**
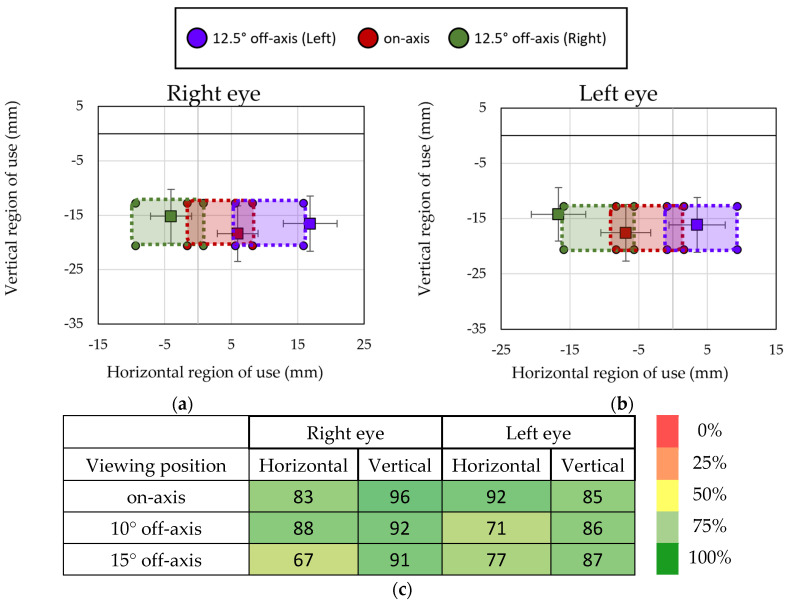
Averaged region of use across subjects and the corresponding standard deviation (in mm) for the right eye (**a**) and the left eye (**b**), represented with squares markers and the theoretical expected range, represented with circle markers and percentage of fixations contained within the expected zone of the lens during distance-reading task (**c**). Data are for all evaluated conditions at distance-reading.

Regarding the concordance between the regions of use, experimentally located and theoretically calculated, results showed an average percentage of concordance of 86% for the located region of use at on-axis viewing position and when rotating the patient at 10° off-axis viewing position. However, the percentage of concordance reduced to 77% at the most peripheral position of 15°. Figure 7a,b shows the mean and standard deviation of the region of use located by the experimental method for the right and left eye for the different viewing positions in comparison with the areas of use of the lens calculated theoretically. The closeness of both data can be seen, suggesting good concordance between both methods. Figure 7c shows the percentage of participants in which all the fixations located by the experimental method were located within the theoretical regions of use. The concordance was higher for on-axis viewing positions and for the identified vertical region in comparison with the horizontal region.

### 3.3. Near-Reading Task

**Figure 8:**
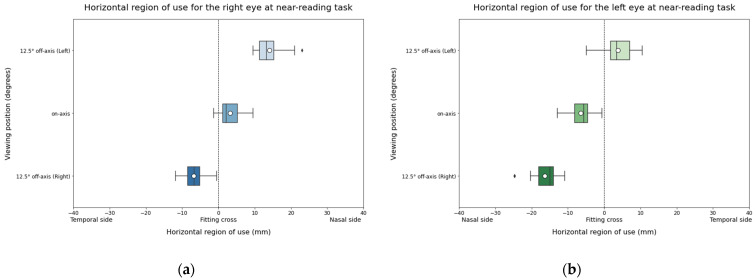
Horizontal region of use for the right eye (**a**) and the left eye (**b**) during NR task. The solid black line represents the median and the circle represents the mean for each box.

Concerning the horizontal region of use, it was expected to find horizontal variations when participants looked through off-axis viewing positions compared to the centered viewing position for both eyes. Results of the study confirmed that when subjects looked at the right column, the left eye shifted towards nasal values while the right eye shifted to temporal values. Similarly, when subjects were reading the left column, the left eye shifted to temporal values while the right eye shifted to nasal values (Figure 8). On the other hand, a nasal shift for the on-axis condition was also expected due to convergence. Comparing data from distance and near reading tasks at on-axis viewing positions (Figure 5 and Figure 8), this displacement of both eyes to the nasal side can be confirmed, indicating that the experimental method is able to evaluate the different areas of use of the lens because of convergence.

**Figure 9:**
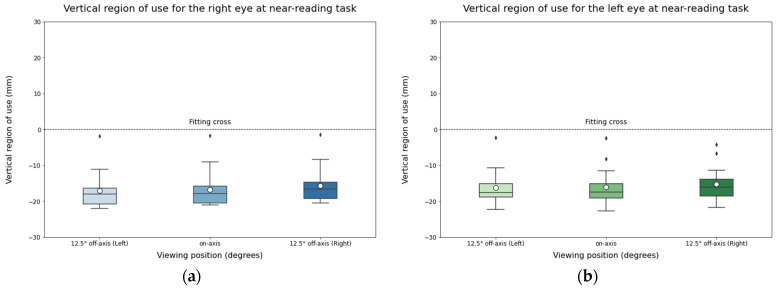
Vertical region of use for the right eye (**a**) and the left eye (**b**) during near-reading task. The solid black line represents the median and the circle represents the mean for each box.

Similar to the distance-reading task, no differences were expected for the vertical region of use, since the test and participants’ head position remained in a stable vertical position across viewing positions (Figure 9).

**Figure 10:**
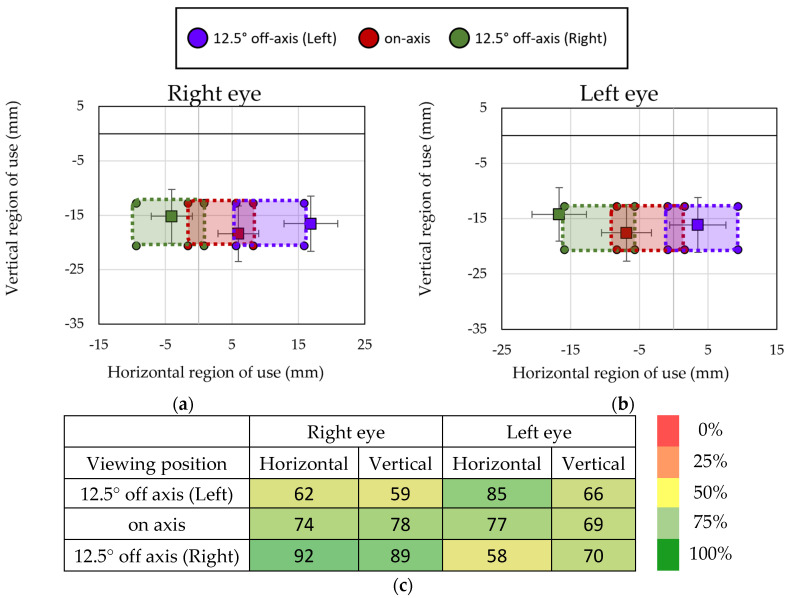
Averaged region of use across subjects and the corresponding standard deviation (in mm) for the right eye (**a**) and the left eye (**b**), represented by square markers, and the theoretical expected range, represented by circle markers, and percentage of fixations contained within the expected zone of the lens during near-reading task (**c**). Data are for all evaluated condition at near-reading.

Regarding the concordance between the experimental and theoretical methods to identify the regions of use, an average percentage of concordance of 73% for the located region of use at on-axis viewing position was obtained. When rotating the patient at an off-axis viewing position, the percentage of concordance was 67% for the left side and 77% for the right side. Figure 10a,b shows the mean and standard deviation of the region of use located by the experimental method for the right and left eye for the different viewing positions in comparison to the theoretical regions geometrically calculated, suggesting that the experimental method provide a good identification of the regions of use of the lens. Figure 10c shows the percentage of participants in which all the fixations located by the experimental method were located within the theoretical regions of use.

## 4. Discussion

The proposed experimental method identifies the region of the lens used during vision-based tasks. In this work, we applied an algorithm in a reading-based experiment, and the results showed variations in the horizontal region of use when participants were induced to look through different viewing positions, both for distance and near reading tasks. Also, the concordance between the regions of use of the lens located by the proposed experimental method and the regions of use of a PPL theoretically calculated were studied, obtaining a correct concordance between both methods.

The set-up of the experiment was designed using a chinrest that forced the participants to look through a specific viewing position and allowed to calculate the expected regions of use in a controlled manner. Regarding the distance-reading task and the horizontal region of use, at off-axis positions, the right eye shifted temporally and the left eye shifted nasally compared to the on-axis viewing position. This shift intensified at 15° off-axis in comparison with 10° off-axis. The eye shift was due to participants’ counterclockwise rotation and the screen’s fixed position. As eccentricity increased relative to the center viewing position, the right eye moved towards the temporal side and the left eye towards the nasal side to look at the screen location. Actual regions of use in the horizontal and vertical axes obtained using the proposed experimental method were compared with the theoretical locations, and it was found that for the distance-reading task, 89% of the fixations were contained within the expected ranges for centered viewing position, 84% of them for 10° off-axis, and 81% of them for 15° off-axis. Concerning the near-reading task and the horizontal region of use, differences were found between the different viewing positions for the right and left eye. While reading the right-column text, participants had a temporal shift in the right eye and a nasal shift in the left eye, compared to the centered position. Conversely, reading the left-column text resulted in a nasal shift in the right eye and a temporal shift in the left eye, differing from the central column reading position. This horizontal eye shift was because of the fixed screen and the use of a chinrest to restrict head movement, influencing the horizontal pupil position for each column. After comparing the experimental value with the expected one, the concordance between them for the near-reading task was 68%, 75%, and 77% at left-side, centered, and right-side viewing positions, respectively.

**Figure 11:**
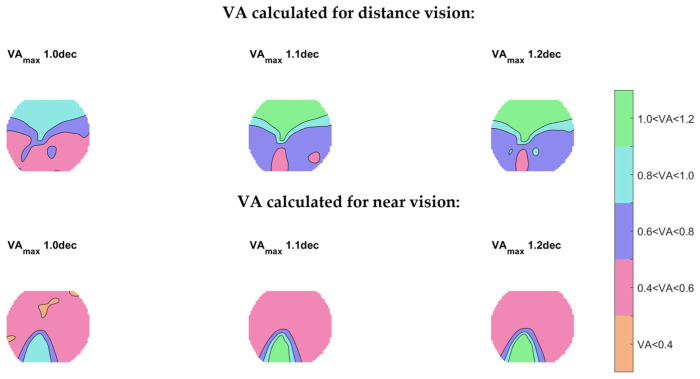
Calculated VA maps for a plano addition 2 D prescription of the Endless Steady Balance (IOT, Spain) lens design achieved by people for different values of maximum VA at distance and near vision using Alonso et al.’s method [17]. The calculation was performed considering an age of 56 years and fusional reserve of ±0.75 D.

The use of a chinrest forced participants to look through regions of the PPL that were not necessary their preferred ones, making wearers use the lateral regions of the PPL and thus allowing the evaluation of off-axis viewing positions. Although these lateral regions were not the ones that would be used if the head were free to move, studies have shown that wearers do not always use the regions of the PPL that are theoretically designed for a specific working distance or task. A study from Cleva et al. [20], investigated PPL use on computer screens in nine presbyopic subjects who wore marked PPL glasses. Participants were asked to read several texts with different letter sizes, vertical positions, and varying distances on a computer and laptop. Results show that users with enough accommodation amplitude do not use the near region, and they tend to use the distance region mainly for higher letter size and higher text position. Participants with a lower accommodation amplitude mainly used the intermediate region of the lens, while the near region was only used with the lower-positioned text screen and smaller letter size. This study supported that wearers might use the regions of the PPL differently than predicted theoretically [6]. In our study, the reading text presented to the participant for distance and near tasks had a size of text that was big enough to allow the participant to read using the lateral region of the lens (0.4 logMAR). Figure 11 shows VA maps for a Plano addition 2D prescription lens design, calculated using the method proposed by Gómez-Pedrero and Alonso. [25], for the same PPL design used in this work. Figure 11 shows that for a stimulus size of 0.4logMAR, participants could use both the central and lateral regions of the lens. Therefore, these results are in line with previous studies, demonstrating that wearers can use areas of the PPL that do not provide the best VA.

Another factor in the experimental set-up was the position of the lenses. They were mounted on a custom clip-on frame attached to eye-tracker glasses, as previously described [17]. This ensured that the eye-tracker data could be recorded gaze data without obstruction. Also, this set-up eliminated the lens effects, such as prismatic distortion or magnification, which would make necessary ray-tracing calculations if the lens were positioned between the participant’s eye and the eye-tracker cameras. However, there are factors that could affect the theoretical identification of the fixation position on the back surface of the lens. On one hand, the location was calculated considering the average pupil diameter of all participants for each task (3.73 and 2.88 mm at distance-reading and near-reading, respectively), even though participants’ pupil sizes reached 5.22 mm and 4.20 mm at distance-reading and near-reading, respectively, according to eye-tracker measurements. This means the location may be more restrictive for those participants with a larger pupil size.

Apart from the pupil size, it is important to have in mind other actors that could play a role in the performance of the algorithm. On the one hand, even though a chinrest system was used to block head movement, the system allows small movements of the patient that may affect the results, which could explain the lower percentage of agreement during off-axis viewing positions (Figure 7c and Figure 10c). This could be considered in the algorithm, considering the gyroscope data that the eye-tracker provides. On the other hand, the eye-tracker device has intrinsic accuracy and precision. In this case, the mean accuracy was 0.6° and the mean precision was 0.03°, which corresponds to 0.34 mm and 0.02 mm on the back surface of the lens, respectively. It is also important to consider other factors that decrease the accuracy and precision of the head-mounted eye-trackers which have been previously analyzed [26], such us extreme viewing positions and reading aloud. In our experiment, we studied each eye monocularly and found a loss of precision mainly from the nasal side during near reading. On the other hand, the proposed experimental method did not consider the lens tilt relative to the eye, pantoscopic angle, or frame wrap angle.

In addition to the current study, our research group has carried out additional studies to determine the precision of the proposed experimental method for identifying regions of use of a progressive power lens. A pilot study in a group of participants was carried out previously to this work, using the same eye-tracker glasses [21]. The left lens of the eye-tracker glasses was occluded, while a pinhole of 1.5 mm diameter was attached to the right lens to force the participants to look through the pinhole. The real position of the pinhole was measured with a millimetric ruler. Participants were asked to look through the pinhole at the target, which was placed in the wall in front of them, while the eye-tracker recorded the pupil’s position. The position determined through the eye-tracker data obtained with the proposed experimental method was compared with the real and known position of the pinhole. Since the left eye was completely covered, only measurements from the right eye were considered for analysis. The pinhole coordinates were contained in the identified pupil positions in all cases; thus, all of them were plausible. In the validity check experiment, horizontal and vertical measurement errors of 1.60 ± 1.23 mm and −0.45 ± 1.44 mm were determined, respectively [21]. Regarding the current study, the error of the pilot study was assumable, since the pupil diameter (3.37 ± 0.62 mm) is more limiting than the measurement error.

Once the proposed experimental method has been validated, it would be interesting to apply it in natural conditions to study lens use and the effect of VA on lens use without a chinrest. Also, it would be interesting to study the regions of use at several working distances. This research only studied distance and near working distances, but PPLs could have different performances at different working distances. In addition, the incorporation of customization parameters could improve accuracy in the calculation of the region of use of the PPL.

Analyzing lens regions via eye-tracker metrics can refine theoretical methods that overlook practical factors. Furthermore, the developed algorithm can be applied to any wearable eye-tracker device that provides gaze direction vector information. In addition, the use of the algorithm can be extended to other ophthalmic lenses. This approach can provide valuable insights for lens designers, helping to identify areas for improvement and enhance wearer comfort and visual experience. Also, this development can be applied by eye care professionals to determine the power most appropriate for each subject, combined with other optical metrics that allow for better lens selection, providing improved ergonomics and visual performance.

## 5. Conclusions

In conclusion, the proposed experimental method identifies the region of the lens used during vision-based tasks. The algorithm has demonstrated the ability to locate regions of the lens compared to expected values. This information could be used to identify the areas of the lens that are really being used, providing insights into how the users’ posture affects lens use, and therefore, helping to design better and more customized progressive lenses.

## Figures and Tables

**Table 1:** Linguistic characteristics of the text used for reading assessment for both distance and near tasks.

Task	Word Count	Syllable Count	Single-Syllable Word Count	Mean Characters per Word	ISFZ Index
Distance-reading	43 ± 2	82 ± 3	19 ± 1	4.4 ± 0.2	84 ± 7
Near-reading	32 ± 2	60 ± 3	13 ± 2	4.4 ± 0.3	85 ± 6

## Data Availability

The data presented in this study are available on request from the corresponding authors.
